# Impact of Intracranial Volume and Brain Volume on the Prognostic Value of Computed Tomography Perfusion Core Volume in Acute Ischemic Stroke

**DOI:** 10.3390/jcdd11030080

**Published:** 2024-02-28

**Authors:** Jan W. Hoving, Praneeta R. Konduri, Manon L. Tolhuisen, Miou S. Koopman, Henk van Voorst, Laura M. Van Poppel, Jasper D. Daems, Adriaan C. G. M. van Es, Marianne A. A. van Walderveen, Hester F. Lingsma, Diederik W. J. Dippel, Wim H. Van Zwam, Henk A. Marquering, Charles B. L. M. Majoie, Bart J. Emmer

**Affiliations:** 1 Department of Radiology and Nuclear Medicine, Amsterdam University Medical Center, University of Amsterdam, 1105 AZ Amsterdam, The Netherlands; m.l.tolhuisen@amsterdamumc.nl (M.L.T.); h.vanvoorst@amsterdamumc.nl (H.v.V.); l.m.vanpoppel@amsterdamumc.nl (L.M.V.P.); h.a.marquering@amsterdamumc.nl (H.A.M.); c.b.majoie@amsterdamumc.nl (C.B.L.M.M.); b.j.emmer@amsterdamumc.nl (B.J.E.); 2 Department of Biomedical Engineering and Physics, Amsterdam University Medical Center, University of Amsterdam, 1105 AZ Amsterdam, The Netherlands; 3 Department of Public Health, Erasmus University Medical Center, P.O. Box 2040 Rotterdam, The Netherlands; 4 Department of Neurology, Erasmus University Medical Center, P.O. Box 2040 Rotterdam, The Netherlands; h.lingsma@erasmusmc.nl (H.F.L.); d.dippel@erasmusmc.nl (D.W.J.D.); 5 Department of Radiology, Leiden University Medical Center, 2333 ZA Leiden, The Netherlands; a.c.g.m.van_es@lumc.nl (A.C.G.M.v.E.); m.a.a.van_walderveen@lumc.nl (M.A.A.v.W.); 6 Department of Radiology and Nuclear Medicine, Cardiovascular Research Institute Maastricht (CARIM), Maastricht University Medical Center+, 6202 AZ Maastricht, The Netherlands; w.van.zwam@mumc.nl

**Keywords:** CT perfusion, stroke, thrombectomy

## Abstract

Background: Computed tomography perfusion (CTP)-estimated core volume is associated with functional outcomes in acute ischemic stroke. This relationship might differ among patients, depending on brain volume. Materials and Methods: We retrospectively included patients from the MR CLEAN Registry. Cerebrospinal fluid (CSF) and intracranial volume (ICV) were automatically segmented on NCCT. We defined the proportion of the ICV and total brain volume (TBV) affected by the ischemic core as ICV_core_ and TBV_core_. Associations between the core volume, ICV_core_, TBV_core_, and functional outcome are reported per interquartile range (IQR). We calculated the area under the curve (AUC) to assess diagnostic accuracy. Results: In 200 patients, the median core volume was 13 (5–41) mL. Median ICV and TBV were 1377 (1283–1456) mL and 1108 (1020–1197) mL. Median ICV_core_ and TBV_core_ were 0.9 (0.4–2.8)% and 1.7 (0.5–3.6)%. Core volume (acOR per IQR 0.48 [95%CI 0.33–0.69]), ICV_core_ (acOR per IQR 0.50 [95%CI 0.35–0.69]), and TBV_core_ (acOR per IQR 0.41 95%CI 0.33–0.67]) showed a lower likelihood of achieving improved functional outcomes after 90 days. The AUC was 0.80 for the prediction of functional independence at 90 days for the CTP-estimated core volume, the ICV_core_, and the TBV_core_. Conclusion: Correcting the CTP-estimated core volume for the intracranial or total brain volume did not improve the association with functional outcomes in patients who underwent EVT.

## 1. Introduction

Computed tomography perfusion (CTP) allows for the quantification of the perfusion status of brain tissue in patients with acute ischemic stroke [1]. The CTP-estimated ischemic core volume is associated with functional outcomes [2,3]. However, accurately predicting functional outcomes for individual patients with acute ischemic stroke remains challenging. Patient-specific brain imaging characteristics, such as intracranial volume (ICV) and total brain volume (TBV), are associated with outcome and may influence the association between the ischemic core volume and functional outcome [4,5]. Brain atrophy—which is characterized by a decrease in TBV due to the loss of brain cells and intercellular connections—is commonly considered when assessing outcomes in qualitative and quantitative neuroimaging research and is associated with functional outcomes after endovascular treatment (EVT) in patients with acute ischemic stroke [6,7,8,9]. Previous studies have shown that the degree of cerebral atrophy—which affects the total brain volume (TBV)—is significantly and independently associated with functional outcomes after EVT [6,7,8]. In addition, it has been shown that a ratio of the CTP-estimated core volume to CSF volume more accurately predicts malignant middle cerebral artery infarction [10]. Differences in ICV exist between different ethnic populations, gender, and age groups [11]. Furthermore, TBV may be affected by restricted CSF absorption (i.e., hydrocephalus), medication use, previous stroke, neurodegenerative diseases, and age itself [12]. In this study, we aim to investigate whether the association between the CTP-estimated ischemic core and functional outcome at 90 days can be improved by correcting the CTP-estimated ischemic core for the ICV or TBV.

## 2. Materials and Methods

### 2.1. Patient Selection

**Figure 1:**
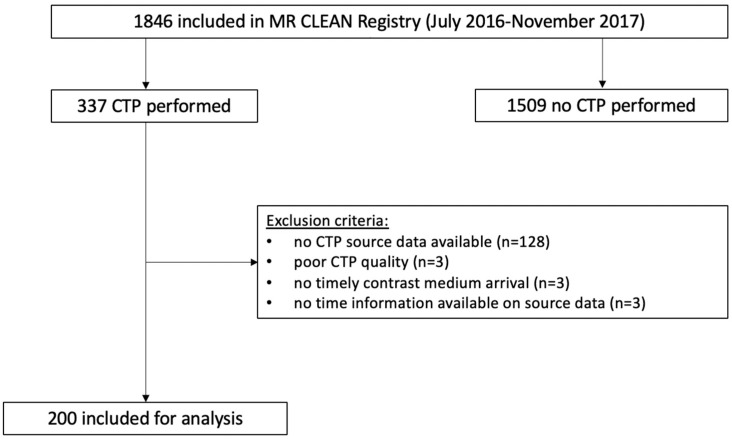
Flowchart of patient selection.

We retrospectively included patients with proximal large vessel occlusion of the anterior cerebral circulation and available baseline CTP source data were included in the MR CLEAN Registry between July 2016 and November 2017. The MR CLEAN Registry is an observational, prospective registry of all consecutive patients undergoing EVT for acute ischemic stroke in the Netherlands [13]. Patients were excluded if CTP source data could not be processed by the CTP analysis software (syngo.via, version VB40) due to motion artifacts or the inadequate caption of contrast medium arrival (Figure 1).

### 2.2. Baseline Image Acquisition, Post-Processing, and Quality Assessment

CTP acquisition was performed according to site-specific protocols. CTP data were centrally processed using syngo.via CT Neuro Perfusion software (version VB40, Siemens Healthineers, Forchheim, Germany). Ischemic core and penumbra were defined as CBV < 1.2 mL/100 mL/s and CBF < 27 mL/100 mL/min, respectively. A default smoothing filter was applied [14]. The CTP results were visually checked by two experienced readers (>5 years of experience). Recanalization success was scored based on the extended thrombolysis in cerebral infarction (eTICI) score on post-treatment digital subtraction angiography (DSA) and ranged from 0 (no antegrade recanalization) to 3 (complete antegrade recanalization) [15].

### 2.3. ICV and Cerebrospinal Fluid (CSF) Assessment

**Figure 2:**
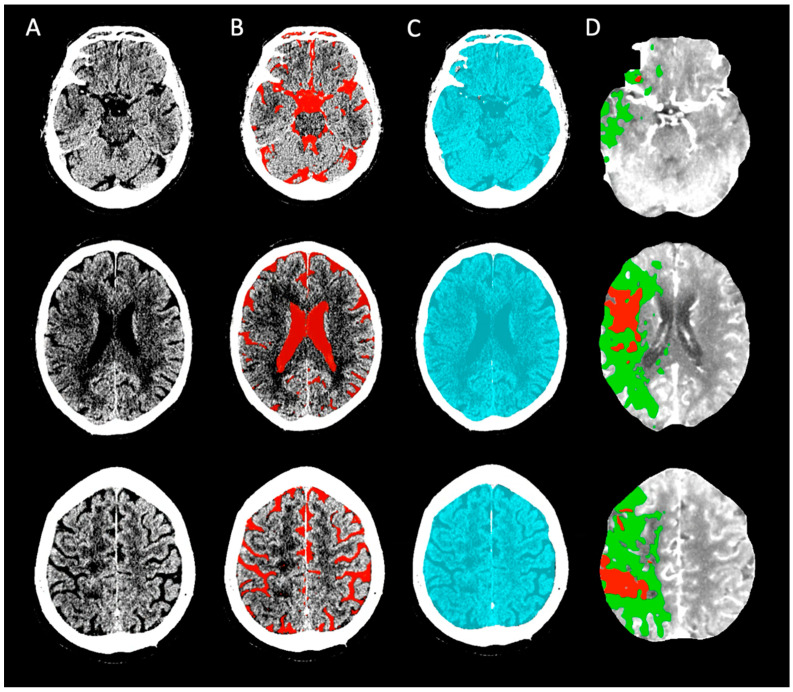
Example of baseline CT imaging with ICV and CSF volume assessments; three levels in the brain are shown. (**A**) Non-contrast CT of a 71-year-old patient with a right-sided M1 occlusion who received IV alteplase before EVT (eTICI 2b). (**B**) CSF segmentation (red) shows a CSF volume of 250 mL. (**C**) ICV segmentation (blue) on baseline NCCT shows an ICV of 1374 mL. (**D**) CTP-estimated core volume (red) was 50 mL. Penumbral volume (green) was 210 mL. CSF = cerebrospinal fluid; CTP = CT perfusion; eTICI = expanded treatment in cerebral infarction; EVT = endovascular treatment; and ICV = intracranial volume.

Baseline NCCT images were post-processed using an automated segmentation algorithm (https://github.com/WCHN/CTseg, accessed on 4 July 2021). This algorithm performed the spatial normalization of the CT images and automatically segmented the CSF volume after skull stripping of the image using a Bayesian approach [16]. ICV was segmented as the complete volume within the skull on baseline NCCT. CSF segmentations were visually checked by an expert neuroradiologist (>15 years of experience) (Figure 2). We determined the ICV and CSF volumes by multiplying the total number of voxels in the segmented intracranial area and CSF with the size of the image voxels, respectively. We calculated TBV by subtracting the CSF volume from ICV. The adjusted CTP-estimated core volumes as a proportion of ICV and TBV were defined as ICV_core_ and TBV_core_ and reported as percentages.

### 2.4. Statistical Analyses

The primary outcome was the 90-day functional outcome scored on the ordinal modified Rankin Scale (mRS) [17]. The secondary outcome was 90-day functional independence (mRS 0–2). We report the crude (cOR) and adjusted common odds ratio (acOR) with 95% confidence intervals (95% CI) for a shift towards improved functional outcomes on the 90-day mRS. We used uni- and multivariable binary and ordinal logistic regression to assess the associations of CTP-estimated core volume, ICV_core_, and TBV_core_ with functional outcome. We identified age, gender, pre-stroke mRS, onset-to-groin time, the administration of intravenous thrombolysis, and baseline NIHSS as potential confounders. Since we measured and calculated continuous variables on different units and scales (i.e., milliliters and percentages), we standardized the odds ratios for ischemic core volume, ICV_core,_ and TBV_core_ by calculating the odds ratio (OR) per interquartile range. The ORs for crude ICV and TBV are presented per 10 mL. We calculated (Tjur’s and Nagelkerke’s) pseudo R^2^ and calculated log-likelihood to determine which model best fits the data. We performed receiver operating characteristic (ROC) analyses to determine the diagnostic accuracy of the unadjusted and adjusted CTP core variables, and the area under the curve (AUC) results were reported. Patients with missing CTP or outcome variables were excluded from our analyses. A *p*-value < 0.05 was considered statistically significant. Statistical analyses were performed in R (R, V3.6.0, R: A language and environment for statistical computing, R Foundation for Statistical Computing, Vienna, Austria).

### 2.5. Protocol Approval and Patient Consent

The Central Medical Ethics Committee of the Erasmus MC, Rotterdam, Netherlands, granted permission to carry out the MR CLEAN Registry (MEC-2014-235). The Ethics Committee waived the requirement of written informed consent for participation.

### 2.6. Data Availability

The datasets presented in this article are not readily available since individual patient data cannot be made available under Dutch law if no consent is obtained. All syntax files are available from the corresponding author upon reasonable request.

## 3. Results

We included 200 patients. A schematic representation of the patient selection is shown in Figure 1. The median age was 71 (IQR 56–80) years, and most patients were men (59%). The median ischemic core volume was 13 (IQR 5–41) mL. The median ASPECTS was 8 (IQR 9–10), the median ICV was 1377 (IQR 1283–1456) mL, the median TBV was 1108 (IQR 1020–1197) mL, the median ICV_core_ was 0.9% (IQR 0.4–2.8%) and the median TBV_core_ was 1.2 (IQR 0.5–3.6) %. Successful recanalization was achieved in 136 (71%) patients. Ninety (48%) patients were functionally independent at 90 days. A detailed overview of the baseline characteristics and outcome is given in Table 1. Pre-stroke mRS and NIHSS at the baseline were not available for 6 (3%) and 3 (2%) patients, respectively. Onset-to-groin time was not available for four (2%) patients. Fourteen (7%) patients were lost to follow-up and had missing outcome variables.

### 3.1. Associations between CTP-Estimated Core Volume, ICV, TBV, and Functional Outcome

The CTP-estimated core volume (cOR per IQR [mL] 0.48 [95%CI 0.33–0.69]), ICV_core_ (cOR per IQR [%] 0.51 [95%CI 0.39–0.69]), and TBV_core_ (cOR per IQR [%] 0.50 [95%CI 0.38–0.67]) were associated with a lower likelihood of improved functional outcomes at 90 days in univariable analyses. TBV was associated with improved functional outcomes (cOR per 10 mL 1.03 [95%CI 1.01–1.05]), whereas ICV was not (cOR per 10 mL 1.01 [95%CI 0.99–1.03]). After adjusting for confounders, the CTP-estimated core volume (acOR per IQR [mL] 0.48 [95%CI 0.33–0.69]), ICV_core_ (acOR per IQR [%] 0.50 [95%CI 0.35–0.69]), and TBV_core_ (acOR per IQR [%] 0.41 [95%CI 0.33–0.67]) were associated with improved functional outcomes. After adjusting for confounders, we did not observe a significant association between either TBV or ICV and improved functional outcomes. ICV and TBV were not statistically significantly associated with the CTP-estimated core volume. Detailed results, including log-likelihood and R^2^ values from the multivariable analyses for CTP-estimated core volume, ICV_core_, and TBV_core_, are provided in Supplementary Table S1 (Supplementary Materials).

### 3.2. Associations between CTP-Estimated Core Volume, ICV, TBV, and Functional Independence

**Figure 3:**
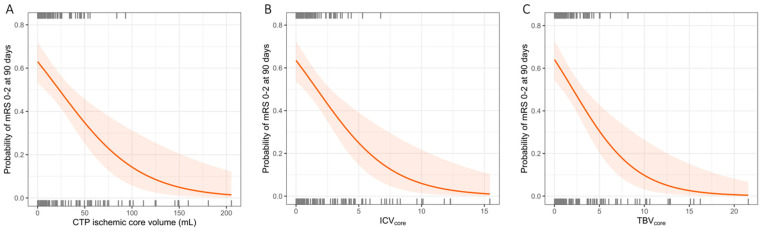
Plot showing the association of (**A**) CTP ischemic core volume, (**B**) the proportion of ICV affected by the CTP ischemic core (ICV_core_), and (**C**) the proportion of TBV affected by the CTP ischemic core (TBV_core_) with the probability of achieving functional independence (mRS 0–2) at 90 days.

**Figure 4:**
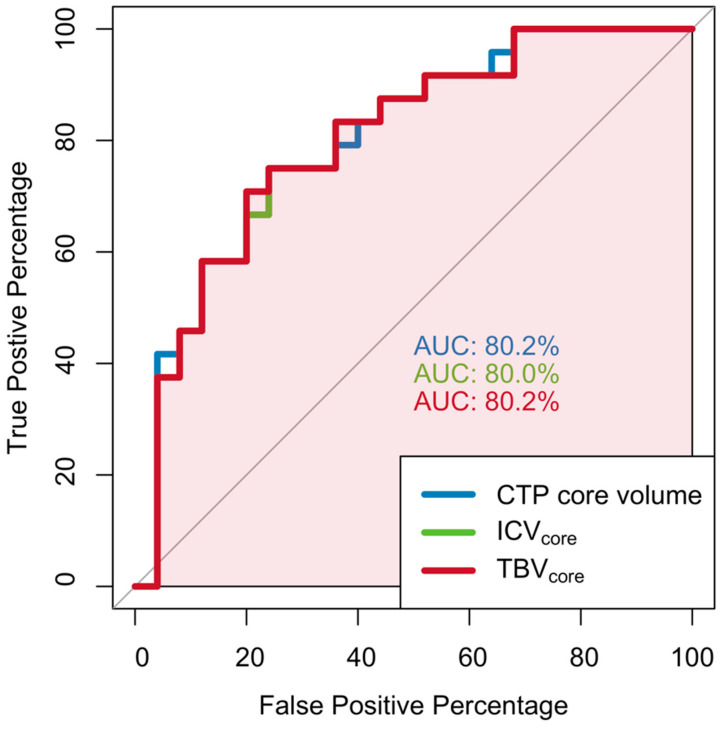
Receiver operating characteristic (ROC) curve for prediction of functional independence (i.e., mRS 0–2) at 90 days based on CTP-estimated core volume (blue line), TBVcore (green line), and ICVcore (red line). CTP = computed tomography perfusion; ICV = intracranial volume; and TBV = total brain volume.

CTP-estimated core volume (cOR per IQR 0.49 [95%CI 0.23–0.70], *p* < 0.001), TBV (OR per IQR 1.10 [95%CI 1.03–1.20]), ICV_core_ (cOR per IQR 0.45 [95%CI 0.28–0.65]) and TBV_core_ (cOR per IQR 0.43 [95%CI 0.26–0.63], *p* < 0.001) were associated with functional independence at 90 days in univariable analysis. The associations between the CTP-estimated ischemic core volume, ICV_core_, and TBV_core_ and functional independence at 90 days are shown in Figure 3. After adjusting for potential confounders, these associations persisted for the CTP-estimated core volume (acOR per IQR 0.34 [95%CI 0.16–0.70]), ICV_core_ (acOR per IQR 0.36 [95%CI 0.19–0.65], and TBV_core_ (acOR per IQR 0.35 [95%CI 0.17–0.63], *p* = 0.002). Details of the multivariable regression analyses for the functional independence of the CTP-estimated core volume, ICV_core_, and TBV_core_ are provided in Supplementary Table S2 of the Supplemental Materials. ROC analysis showed an AUC of 0.80 for the prediction of functional independence at 90 days for the CTP-estimated core volume, ICV_core_, and TBV_core_. The results of the ROC analysis are shown in Figure 4.

## 4. Discussion

This post hoc analysis of the MR CLEAN Registry showed that correcting the CTP-estimated ischemic core volume for the ICV or TBV did not result in improved functional outcome predictions compared to the CTP-estimated core volume alone. The ROC analyses showed similar diagnostic performance for all prognostic models in terms of the AUC. This could be explained by the fact that the AUC is relatively insensitive to the additional contribution of a biometric when this is estimated on a continuous scale, especially when the investigated models contain the same biometric in adjusted and unadjusted forms [18]. Therefore, it has been suggested that ORs obtained from regression analyses are more useful for explaining associations of (imaging) metrics with clinical events, such as functional independence at 90 days in patients with acute ischemic stroke [18].

Previous studies showed that both the CTP-estimated core volume and—surrogates of—brain atrophy are associated with functional outcomes after EVT [3,6,7,19,20,21,22]. For example, a post hoc analysis from the MR CLEAN trial found that cerebral atrophy modifies the effect of EVT and that the benefit of EVT was larger in patients with more severe atrophy [20]. Another retrospective cohort study showed that an increased CSF volume, as an imaging marker for biological brain age, was associated with a reduced likelihood of achieving functional independence at 90 days in patients who underwent EVT [6]. Two recent MRI-based studies confirmed this by demonstrating that TBV is an important prognostic marker of functional outcomes after stroke [4,22]. In line with these studies, we found that TBV was associated with functional outcomes. However, since none of the previous studies on the effect of brain atrophy considered CTP results, the question of whether brain imaging metrics provide additional information assessing the CTP-estimated ischemic core volume cannot be answered yet. Our observation that the association between CTP-estimated ischemic core and functional outcome was not improved by determining the proportion of affected ICV or TBV confirms—at least in part—that the relationships between baseline (imaging) characteristics and functional outcomes in acute ischemic stroke are complex and likely to be multifactorial. For example, although it is generally considered that patients with increased CSF volumes have a larger buffer in the case of edema formation [19], patients with smaller brain volumes (e.g., due to a higher frequency of other cerebrovascular comorbidities) are generally older and have worse functional outcomes, despite increased CSF volumes [23].

Several limitations to this study should be noted. First, since patients in our study cohort had relatively small ischemic core volumes (i.e., median 1% of the ICV) and the variation in ICV was limited, our results are probably not generalizable to populations with larger core volumes or more diverse intracranial volumes. Future studies focusing on the effect of the ischemic core volume in relation to brain volume should restrict their brain volume measurements to the parenchymal volume of a single hemisphere or to the specific affected vascular territory from both the affected and the contralateral hemisphere.

Second, it is important to consider that only data from EVT-treated patients were included in the MR CLEAN Registry, as well as the actual treatment effect; therefore, any potential treatment effect modification by any of the studied imaging metrics could not be measured. Third, we were not able to validate our models on an external cohort. Finally, selection bias might have occurred as patients with poor clinical and imaging profiles might have been excluded from EVT. Similarly, patients with a high clinical suspicion of LVO may not have received CTP imaging and directly underwent EVT. Our findings should be validated in a setting where CTP imaging is routinely performed, preferably including data from patients who did not undergo EVT.

## 5. Conclusions

Correcting the CTP-estimated ischemic core volume for the ICV or TBV does not improve the association with functional outcomes in patients who underwent EVT compared to using the CTP-estimated core volume alone.

## Figures and Tables

**Table 1:** Baseline characteristics and outcome data of the MR CLEAN Registry subpopulation included in this analysis compared to the overall MR CLEAN Registry cohort. ASPECTS = Alberta Stroke Program Early CT Score; CSF = cerebrospinal fluid; CTP = CT perfusion; ICA = intracranial carotid artery; ICA-T = intracranial carotid artery terminus; IVT = IV alteplase; IQR = interquartile range; M1 = M1 segment of the middle cerebral artery; M2 = M1 segment of the middle cerebral artery; mRS = modified Rankin Score; and NIHSS = National Institute of Health Stroke Scale. If the [known in] number is not shown, the variable was known in all patients. * = Time between symptom onset and imaging at a comprehensive stroke center.

	MR CLEAN Registry CTP Subgroup (n = 200)	Overall MR CLEAN Registry (n = 1755)
***Clinical***		
**Age (yr)**—median (IQR)	71 (56–80)	72 (62–81)
**Female**—n (%)	83 (42)	889 (51)
**NIHSS at baseline**—median (IQR) [known in]	16 (12–20) [n = 197]	16 (11–19)
**Transfer from primary stroke center**—n (%)	19 (10)	962 (55)
**IVT administered**—n (%)	144 (72)	1282 (73)
Onset-to-imaging time (min) *—median (IQR) [known in]	79 (56–137) [N = 194]	76(53–128) [N = 1279]
**Onset-to-groin time (min)**—median (IQR) [known in]	153 (120–223) [N = 196]	185(144–243) [N = 1740]
***Imaging***		
**Occlusion location on baseline CTA**—n (%) [known in] *Intracranial ICA* *ICA-T* *M1* *M2* *Other*	[N = 198] 6 (3) 35 (18) 121 (61) 35 (18) 1 (1)	[N = 1657] 76 (4) 342 (20) 974 (56) 295 (17) 6 (0.3)
**ASPECTS**—median (IQR) [known in]	9 (8–10) [N = 199]	9 (8–10) [N = 1713]
**Collateral status**—n (%) [known in] *0* *1* *2* *3*	[N = 197] 8 (4) 79 (40) 82 (42) 28 (14)	[N = 1693] 89 (5) 635 (36) 643 (37) 290 (17)
**Baseline ischemic core volume on CTP (mL)**—median (IQR)	13 (5–41)	NA
**Baseline penumbra volume on CTP (mL)**—**median (IQR)**	96 (58–123)	NA
**Intracranial volume (ICV) (mL)**—median (IQR) Males Females	1377 (1283–1456) 1435 (1378–1502) 1280 (1224–1330)	NA
**Total brain volume (TBV) (mL)**—**median (IQR)** Males Females	1109 (1020–1196) 1170 (1106–1233) 1020 (976–1082)	NA
**CSF volume (mL)**—median (IQR) Males Females	258 (229–296) 266 (237–303) 246 (216–286)	NA

## Data Availability

The datasets presented in this article are not readily available since individual patient data cannot be made available under Dutch law if no consent is obtained. All syntax files are available from the corresponding author upon reasonable request.

## Supplementary Materials

The following supporting information can be downloaded at: https://www.mdpi.com/article/10.3390/jcdd11030080/s1, Table S1: Multivariable analysis of improved functional outcomes (mRS) at 90 days; Table S2: Multivariable analysis of functional independence (mRS 0–2).

## Appendix A

MR CLEAN Registry investigators:

Executive committee

Diederik W.J. Dippel^1^; Aad van der Lugt^2^; Charles B.L.M. Majoie^3^; Yvo B.W.E.M. Roos^4^; Robert J. van Oostenbrugge^5,44^; Wim H. van Zwam^6,44^; Jelis Boiten^14^; Jan Albert Vos^8^

Study coordinators

Ivo G.H. Jansen^3^; Maxim J.H.L. Mulder^1,2^; Robert- Jan B. Goldhoorn^5,6,44^; Kars C.J. Compagne^2^; Manon Kappelhof^3^; Josje Brouwer^4^; Sanne J. den Hartog^1,2,40^; Wouter H. Hinsenveld^5,6^; Lotte van den Heuve^l1,40^.

Local principal investigators

Diederik W.J. Dippe^l1^; Bob Roozenbeek^1^; Aad van der Lugt^2^; Pieter Jan van Doormaal^2^, Charles B.L.M. Majoie^3^; Yvo B.W.E.M. Roos^4^; Bart J. Emmer^3^; Jonathan M. Coutinho^4^; Wouter J. Schonewille^7^; Jan Albert Vos^8^; Marieke J.H. Wermer^9^; Marianne A.A. van Walderveen^10^; Adriaan C.G.M. van Es^10^; Julie Staals^5,44^; Robert J. van Oostenbrugge^5,44^; Wim H. van Zwam^6,44^; Jeannette Hofmeijer^11^; Jasper M. Martens^12^; Geert J. Lycklama à Nijeholt^13^; Jelis Boiten^14^; Sebastiaan F. de Bruijn^15^; Lukas C. van Dijk^16^; H. Bart van der Worp^17^; Rob H. Lo^18^; Ewoud J. van Dijk^19^; Hieronymus D. Boogaarts^20^; J. de Vries^22^; Paul L.M. de Kort^21^; Julia van Tuij^l21^; Issam Boukrab^26^; Jo P. Peluso^26^; Puck Fransen^22^; Jan S.P. van den Berg^22^; Heleen M. den Hertog^22^; Boudewijn A.A.M. van Hasselt^23^; Leo A.M. Aerden^24^; René J. Dallinga^25^; Maarten Uyttenboogaart^28^; Omid Eschgi^29^; Reinoud P.H. Bokkers^29^; Tobien H.C.M.L. Schreuder^30^; Roel J.J. Heijboer^31^; Koos Keizer^32^; Rob A.R. Gons^32^; Lonneke S.F. Yo^33^; Emiel J.C. Sturm^35,47^, Tomas Bulut^35^; Paul J.A.M. Brouwers^34^; Anouk D. Rozeman^42^; Otto Elgersma^41^, Michel J.M. Remmers^43^; Thijs E.A.M. de Jong^46^.

Imaging assessment committee

Charles B.L.M. Majoie^3^(chair); Wim H. van Zwam^6,44^; Aad van der Lugt^2^; Geert J. Lycklama à Nijeholt^13^; Marianne A.A. van Walderveen^10^; Marieke E.S. Sprengers^3^; Sjoerd F.M. Jenniskens^27^; René van den Berg^3^; Albert J. Yoo^38^; Ludo F.M. Beenen^3^; Alida A. Postma^6.45^; Stefan D. Roosendaa^l3^; Bas F.W. van der Kallen^13^; Ido R. van den Wijngaard^13^; Adriaan C.G.M. van Es^10^; Bart J. Emmer,^3^; Jasper M. Martens^12^; Lonneke S.F. Yo^33^; Jan Albert Vos^8^; Joost Bot^36^; Pieter-Jan van Doormaa^l2^; Anton Meijer^27^; Elyas Ghariq^13^; Reinoud P.H. Bokkers^29^; Marc P. van Proosdij^37^; G. Menno Krietemeijer^33^; Jo P. Peluso^26^; Hieronymus D. Boogaarts^20^; Rob Lo^18^;Wouter Dinkelaar^41^; Auke P.A. Appelman^29^; Bas Hammer^16^; Sjoert Pegge^27^; Anouk van der Hoorn^29^; Saman Vinke^20^; Sandra Cornelissen^2^; Christiaan van der Leij^6^; Rutger Brans6; Jeanette Bakker^41^; Maarten Uyttenboogaart^28^; Miou Koopman^3^; Lucas Smagge^2^; Olvert A. Berkhemer^1,3,6^; Jeroen Markenstein^3^; Eef Hendriks^3^; Patrick Brouwer^10^; Dick Gerrits^35^.

Writing committee

Diederik W.J. Dippel^1^(chair); Aad van der Lugt^2^; Charles B.L.M. Majoie^3^; Yvo B.W.E.M. Roos^4^; Robert J. van Oostenbrugge^5,44^; Wim H. van Zwam^6,44^; Geert J. Lycklama à Nijeholt^13^; Jelis Boiten^14^; Jan Albert Vos^8^; Wouter J. Schonewille^7^; Jeannette Hofmeijer^11^; Jasper M. Martens^12^; H. Bart van der Worp^17^; Rob H. Lo^18^

Adverse event committee

Robert J. van Oostenbrugge^5,44^(chair); Jeannette Hofmeijer^11^; H. Zwenneke Flach^23^

Trial methodologist

Hester F. Lingsma^40^

Research nurses / local trial coordinators

Naziha el Ghannouti^1^; Martin Sterrenberg^1^; Wilma Pellikaan^7^; Rita Sprengers^4^; Marjan Elfrink^11^; Michelle Simons^11^; Marjolein Vossers^12^; Joke de Meris^14^; Tamara Vermeulen^14^; Annet Geerlings^19^; Gina van Vemde^22^; Tiny Simons^30^; Gert Messchendorp^28^; Nynke Nicolaij^28^; Hester Bongenaar^32^; Karin Bodde^24^; Sandra Kleijn^34^; Jasmijn Lodico^34^; Hanneke Droste^34^; Maureen Wollaert^5^; Sabrina Verheesen^5^; D. Jeurrissen^5^; Erna Bos^9^; Yvonne Drabbe^15^; Michelle Sandiman^15^; Nicoline Aaldering^11^; Berber Zweedijk^17^; Jocova Vervoort^21^; Eva Ponjee^22^; Sharon Romvie^l19^; Karin Kanselaar^19^; Denn Barning^10^ ; Laurine van der Steen^3^.

Clinical/imaging data aquisition

Esmee Venema^40^; Vicky Chalos1,^40^; Ralph R. Geuskens^3^; Tim van Straaten^19^; Saliha Ergezen^1^; Roger R.M. Harmsma^1^; Daan Muijres^1^; Anouk de Jong^1^; Olvert A. Berkhemer^1,3,6^; Anna M.M. Boers^3,39^; J. Huguet^3^; P.F.C. Groot^3^; Marieke A. Mens^3^; Katinka R. van Kranendonk^3^; Kilian M. Treurniet^3^; Manon L. Tolhuisen^3,39^; Heitor Alves^3^; Annick J. Weterings^3^; Eleonora L.F. Kirkels^3^; Eva J.H.F. Voogd^11^; Lieve M. Schupp^3^; Sabine L. Collette^28,29^; Adrien E.D. Groot^4^; Natalie E. LeCouffe^4^; Praneeta R. Konduri^39^; Haryadi Prasetya^39^; Nerea Arrarte-Terreros^39^; Lucas A. Ramos^39^ ; Nikki Boodt1,^2,40^; Anne F.A.V Pirson^5^; Agnetha A.E. Bruggeman^3^; Nadinda A.M. van der Ende ^1,2^, Rabia Deniz^3^, Susanne G.H. Olthuis^5,44^, Floor Pinckaers^6,44^

List of affiliations

Department of Neurology^1^, Radiology^2^, Public Health^40^, Erasmus MC University Medical Center;

Department of Radiology and Nuclear Medicine^3^, Neurology^4^, Biomedical Engineering & Physics^39^, Amsterdam UMC, location University of Amsterdam;

Department of Neurology^5^, Radiology & Nuclear Medicine^6^, Maastricht University Medical Center+; School for Cardiovascular Diseases Maastricht (CARIM)^44^; and MHeNs School for Mental Health and Neuroscience, Maastricht, the Netherlands^45^;

Department of Neurology^7^, Radiology^8^, Sint Antonius Hospital, Nieuwegein;

Department of Neurology^9^, Radiology^10^, Leiden University Medical Center;

Department of Neurology^11^, Radiology^12^, Rijnstate Hospital, Arnhem;

Department of Radiology^13^, Neurology^14^, Haaglanden MC, the Hague;

Department of Neurology^15^, Radiology^16^, HAGA Hospital, the Hague;

Department of Neurology^17^, Radiology^18^, University Medical Center Utrecht;

Department of Neurology^19^, Neurosurgery^20^, Radiology^27^, Radboud University Medical Center, Nijmegen;

Department of Neurology^21^, Radiology^26^, Elisabeth-TweeSteden ziekenhuis, Tilburg;

Department of Neurology^22^, Radiology^23^, Isala Klinieken, Zwolle;

Department of Neurology^24^, Radiology^25^, Reinier de Graaf Gasthuis, Delft;

Department of Neurology^28^, Radiology^29^, University Medical Center Groningen;

Department of Neurology^30^, Radiology^31^, Atrium Medical Center, Heerlen;

Department of Neurology^32^, Radiology^33^, Catharina Hospital, Eindhoven;

Department of Neurology^34^, Radiology^35^, Medisch Spectrum Twente, Enschede, (currently Deventer Hospital47);

Department of Radiology^36^, Amsterdam UMC, Vrije Universiteit van Amsterdam, Amsterdam;

Department of Radiology^37^, Noordwest Ziekenhuisgroep, Alkmaar;

Department of Radiology^38^, Texas Stroke Institute, Texas, United States of America;

Department of Neurology^42^, Radiology^41^, Albert Schweitzer Hospital, Dordrecht;

Department of Neurology^43^, Radiology^46^, Amphia Hospital, Breda.
