# Supplementary material for: Impact of Intracranial Volume and Brain Volume on the Prognostic Value of Computed Tomography Perfusion Core Volume in Acute Ischemic Stroke
Source: J Cardiovasc Dev Dis. 2024 Feb 28;11(3):80. doi: 10.3390/jcdd11030080 (PMC10971384; doi:10.3390/jcdd11030080)
Supplement: Supplementary file 1 [file jcdd-11-00080-s001.zip › jcdd-2806124-supplementary.pdf]

SUPPPLEMENTAL MATERIAL

Supplemental Table I. Multivariable analysis of improved functional outcome (mRS) at 90 days. ASPECTS, Alberta Stroke Program Early CT Score; intravenous alteplase; mRS, modified Rankin Scale; NIHSS, National Institutes of Health Stroke Scale; aOR, adjusted Odds Ratio.

**Variables**		**aOR(95% CI)**	***P* value**	**Log-likelihood**	**Nagelkerke's R^2**	**β**
**CTP ischemic core volume (per IQR [mL])**		**0.48 (0.33-0.69)**	**<0.001**			-0.21
	**Age (per year)**	**0.97 (0.95-0.99)**	**0.002**			-0.03
	Male gender	1.71 (0.97-3.04)	0.07			0.54
	Onset-to-groin time (per minute)	1.00 (0.99-1.00)	0.10			-0.002
	NIHSS at baseline (per point)	0.96 (0.92-1.01)	0.14			-0.04
	**Pre-stroke mRS**	**0.55 (0.42-0.71)**	**<0.001**			-0.59
	IVT administration	0.81 (0.43-1.153)	0.52			-0.21
				-284.46	0.51	
**ICV_{core} (per IQR [%])**		**0.51 (0.39-0.69)**	**<0.001**			-0.29
	**Age (per year)**	**0.97 (0.95-0.99)**	**0.002**			-0.03
	Male gender	1.63 (0.93-2.89)	0.09			0.49
	Onset-to-groin time (per minute)	1.00 (0.99-1.00)	0.10			-0.002
	NIHSS at baseline (per point)	0.96 (0.93-1.01)	0.14			-0.04
	**Pre-stroke mRS**	**0.55 (0.42-0.71)**	**<0.001**			-0.59
	IVT administration	0.81 (0.43-1.53)	0.52			-0.21
				-284.63	0.51	
**TBV_{core} (per IQR [%])**		**0.50 (0.38-0.67)**	**<0.001**			-0.24
	**Age (per year)**	**0.97 (0.95-0.99)**	**0.002**			-0.03
	Male gender	1.65 (0.94-2.91)	0.09			0.50
	Onset-to-groin time (per minute)	1.00 (0.99-1.00)	0.09			-0.002
	NIHSS at baseline (per point)	0.96 (0.92-1.01)	0.14			-0.04
	**Pre-stroke mRS**	**0.55 (0.42-0.72)**	**<0.001**			-0.59
	IVT administration	0.81 (0.43-1.53)	0.52			-0.21
				-284.21	0.52	

Supplemental Table II. Multivariable analysis of functional independence (mRS 0-2). ASPECTS, Alberta Stroke Program Early CT Score; IVT, intravenous alteplase; mRS, modified Rankin Scale; NIHSS, National Institutes of Health Stroke Scale; aOR , adjusted Odds Ratio.

Variables		aOR(95% CI)	*P* value	Log-likelihood	Tjur's R^2	β
**CTP ischemic core volume (per IQR [mL])**		**0.34 (0.16-0.70)**	**0.001**			-0.30
	**Age (per year)**	**0.94 (0.91-0.97)**	**<0.001**			-0.06
	Male gender	2.09 (0.94-4.75)	0.07			0.74
	Onset-to-groin time (per minute)	1.00 (1.00-1.00)	0.4			-0.001
	NIHSS at baseline (per point)	0.97 (0.90-1.04)	0.3			-0.04
	**Pre-stroke mRS**	**0.38 (0.22-0.60)**	**<0.001**			-0.96
	IVT administration	0.84 (0.34-2.02)	0.7			-0.17
				-79.05	0.41	
**ICV_{core} (per IQR [%])**		**0.36 (0.19-0.65)**	**0.002**			-0.41
	**Age (per year)**	**0.94 (0.91-0.97)**	**<0.001**			-0.06
	Male gender	1.93 (0.87-4.34)	0.1			0.68
	Onset-to-groin time (per minute)	1.00 (1.00-1.00)	0.4			-0.001
	NIHSS at baseline (per point)	0.97 (0.90-1.04)	0.3			-0.04
	**Pre-stroke mRS**	**0.38 (0.22-0.60)**	**<0.001**			-0.96
	IVT administration	0.84 (0.34-2.03)	0.7			-0.17
				-79.35	0.41	
**TBV_{core} (per IQR [%])**		**0.35 (0.17-0.63)**	**0.002**			-0.34
	**Age (per year)**	**0.94 (0.91-0.97)**	**<0.001**			-0.06
	Male gender	1.94 (0.87-4.36)	0.1			0.66
	Onset-to-groin time (per minute)	1.00 (1.00-1.00)	0.4			-0.001
	NIHSS at baseline (per point)	0.97 (0.90-1.04)	0.3			-0.03
	**Pre-stroke mRS**	**0.38 (0.22-0.60)**	**<0.001**			-0.96
	IVT administration	0.84 (0.34-2.03)	0.7			-0.17
				-79.17	0.41	
